# Pathogenic variation types in human genes relate to diseases through Pfam and InterPro mapping

**DOI:** 10.3389/fmolb.2022.966927

**Published:** 2022-09-16

**Authors:** Giulia Babbi, Castrense Savojardo, Davide Baldazzi, Pier Luigi Martelli, Rita Casadio

**Affiliations:** ^1^ Biocomputing Group, Department of Pharmacy and Biotechnology, University of Bologna, Bologna, Italy; ^2^ Centro di Riferimento Oncologico (CRO), Aviana, Italy; ^3^ Institute of Biomembranes, Bioenergetics and Molecular Biotechnologies (IBIOM), Italian National Research Council (CNR), Bari, Italy

**Keywords:** disease associated variant, variation physicochemical type, Pfam domain, InterPro domain, mondo anatomical system categories

## Abstract

Grouping residue variations in a protein according to their physicochemical properties allows a dimensionality reduction of all the possible substitutions in a variant with respect to the wild type. Here, by using a large dataset of proteins with disease-related and benign variations, as derived by merging Humsavar and ClinVar data, we investigate to which extent our physicochemical grouping procedure can help in determining whether patterns of variation types are related to specific groups of diseases and whether they occur in Pfam and/or InterPro gene domains. Here, we download 75,145 germline disease-related and benign variations of 3,605 genes, group them according to physicochemical categories and map them into Pfam and InterPro gene domains. Statistically validated analysis indicates that each cluster of genes associated to Mondo anatomical system categorizations is characterized by a specific variation pattern. Patterns identify specific Pfam and InterPro domain–Mondo category associations. Our data suggest that the association of variation patterns to Mondo categories is unique and may help in associating gene variants to genetic diseases. This work corroborates in a much larger data set previous observations from our group.

## Introduction

Modern sequencing technologies and intensive research on the molecular origins of humans are increasing exponentially the number of missense single-nucleotide mutations leading to observable changes in protein sequences, and evidently, in their function. For many of these single-residue variations (SRVs), links to disease are reported in public databases such as Humsavar^1^ (The UniProt Consortium, 2021), the UniProt dataset of human missense variants, and ClinVar^2^ (Landrum et al., 2018), the NCBI resource of relationships among human variations and disease phenotypes.

In this scenario, harmonisation of disease definition is an issue for a better association of molecular events to phenotypes (McInnes et al., 2021). Recently the Mondo Disease Ontology^3^, in its semi-automatic version that includes also manual curation (Mungall et al., 2017), integrates multiple disease resources to yield a coherent merged ontology. Furthermore, thanks to the interoperability provided by the Ontology Lookup Service (part of the ELIXIR infrastructure^4^), it is now available for browsing^5^, making it feasible to merge data from different databases for a larger inclusion of variations when characterising variant-disease association. Indeed, the relationship between sequence variation and disease predisposition can identify processes that are responsible of pathogenesis and can help in highlighting new treatments (McCarthy and MacArthur, 2017; Claussnitzer et al., 2020; Sheils et al., 2021).

More to this, genome-wide association studies (GWAS) have identified thousands of noncoding loci that are associated with human diseases and complex traits, each of which could reveal insights into the mechanisms of disease. Particularly interesting is the network of genome-wide enhancers, which links variations to target disease genes, recently described (Nasser et al., 2021, and references therein). This stands from the estimation of which enhancers regulate which genes in the genome and the enhancer-promoter contact frequency from epigenomic datasets, supporting the general notion that variations and gene-mediated disease associations are a very complex phenomenon, which occurs at the cell level (Nasser et al., 2021)^6^.

Different methods are available for functional variant annotations, before their depositions in specific databases (Hebbar and Sowmya, 2022). On the other hand, computational methods try to establish rules of association between variations and diseases with the purpose of helping the annotation process of the newly sequenced variants, exomes, and genomes (for recent implementaions see Pei and Grishin, 2021; Woodard et al., 2021, and references therein). Methods rely on inference processes standing upon the knowledge present in databases and require validated sets of variation-disease associations (Glusman et al., 2017; Peng et al., 2019; Sarkar et al., 2020; Vihinen, 2021). Alternatively, other methods based on disease-domain associations and pathway-specific protein domains (Zhang et al., 2016; Shim et al., 2019, respectively) have been proposed.

A major problem in addressing the problem of gene-disease association is that data constantly increase and that the name and/or number of diseases associated to a single gene is strongly depending on which database you are referring to (Grissa et al., 2022). With the increasing amount of available data, we are now interested in understanding to which extent gene structural and functional features may help in relating variations to diseases. For this reason, we decided to focus on structural and functional mapping of genes and their variants with Pfam^7^ and InterPro^8^ domains (Mistry et al., 2021). In a previous study, we found that, in human proteins, pathogenic variations group into variational patterns that differ depending on the Pfam domain and the group of diseases they link (Savojardo et al., 2019; 2021b; 2021a). Here, we extend the analysis to a much larger data set of germline variations generated by the union of Humsavar and ClinVar. Besides Pfam, in this paper we include functional features as described by InterPro domains and find that Pfam and InterPro regions, covering most of the union data set, specifically relate variations to associated diseases. Furthermore, we show that different Mondo categories are associated to different Pfam and InterPro regions in a significant manner, supporting the notion that a specific disease may relate to the gene variant knowing the location of the corresponding variations in specific structural or functional domains.

## Materials and methods

### Data collection

Variations were collected from Humsavar (The UniProt Consortium, 2021)^9^ and ClinVar (Landrum et al., 2018)^10^, along with the annotation of their effect on human health following the classification scheme of the American College of Medical Genetics and Genomics/Association for Molecular Pathology terminology (Richards et al., 2015). In this work, we focus on germline variations, and we identify genes with the corresponding UniProt reference protein. ClinVar adopts a more detailed labelling than Humsavar. For sake of simplicity, ClinVar variations labelled as Likely Pathogenic or Pathogenic (LP/P), Pathogenic (P) and Likely Pathogenic (LP) where merged into a unique LP/P class, like in Humsavar. Similarly Likely Benign or Benign (LB/B), Likely Benign (LB) and Benign (B) where grouped in the class LB/B, following Humsavar. Furthermore, LB/B variations were collected only when associated to genes with disease-related variations. Variations of Uncertain Significance were discarded from both databases.

We collected our dataset, adopting the following procedure.

• From Humsavar (release: 8/04/2021) we collected 30,415 unique single residue variations annotated as LP/P in 3,043 genes and their included LB/B variations; from ClinVar (release: 29/03/2021) we extracted 38,415 missense variations annotated as pathogenic, likely pathogenic or pathogenic/likely pathogenic in 3,842 genes and their included LB, B and LB/B variations. With this, we consider only LB/B variations in disease associated genes.
• Gene variations were mapped on the corresponding UniProt canonical protein sequences by means of the RefSeq transcript (NM) and protein (NP and WP) accessions. We found that 93% of the whole variation set mapped to the UniProt canonical sequence. We checked the consistency between the protein sequence and the wild-type residue of the reported missense variation.
• Somatic variations and variations with contrasting effect in the two databases were discarded.
• Associations of gene variations to specific diseases were retrieved by means of the OMIM disease codes (Amberger et al., 2019) in Humsavar and of the OMIM, Orphanet, HPO, MeSH, and Mondo codes in ClinVar.
• Associated diseases were annotated with the “disease or disorder” branch in the Mondo ontology^11^ (Mungall et al., 2017), apart from 71 OMIM diseases without any IDs in Mondo. All the variations associated to diseases without an OMIM and/or a Mondo ID were discharged.

### Disease classification

We classify diseases following the Mondo “Disease by Anatomical System” categorization, as reported by EMBL-EBI Ontology Lookup Service^12^. According to this Mondo categorization^13^, diseases group in relation to their effects on the functioning of an organ system. For sake of brevity, when necessary, we arbitrarily label the 14 Mondo “Disease by Anatomical System” categories as follows: **A**-respiratory system disease, **B**-auditory system disease, **C**-immune system disease, **D**-digestive system disease, **E**-disease of the genitourinary system, **F**-hematologic disease, **G**-endocrine system disease, **H**-urinary system disease, **I**-integumentary system disease, **J**-cardiovascular disease, **K**-musculoskeletal system disease, **L**-disease of the visual system, **M**-nervous system disorder, **N**-mediastinal disease.

5,223 Mondo IDs are classified in 13 of the 14 Mondo anatomical system categories, except for the “mediastinal disease” anatomical category, which includes only one variation, and it has been therefore excluded from the analysis.

### Pfam and InterPro annotation

Pfam annotations (version 33.1) were downloaded for the human proteome from the Pfam FTP server^14^. Annotations were filtered to retain only those occurring in genes included in our dataset and covering at least one pathogenic SRV.

Analogously, InterPro annotations including all signatures for human genes were extracted from the complete UniProt protein annotation file available in the InterPro website^15^. We retained only InterPro signatures mapping on genes in our set and covering pathogenic SRVs.

### Statistical validation

The significance of the observed difference between Pfam/InterPro-specific distributions of variation types and Mondo anatomical system categories against respective background distributions has been assessed using an FDR-corrected Chi-squared test. Given a domain-specific observed counts $c_{o}=\left(c_{o}^{1},\cdots ,c_{o}^{K}\right)$ for *K* possible events (either counting SRV types or Mondo categories) and a corresponding background distribution $f_{b}=\left(f_{b}^{1},\cdots ,f_{b}^{K}\right)$ , we compute the Chi-squared test statistics as:

$$\chi^{2}=\overset{K}{\underset{i=1}{\sum }}\frac{{\left(c_{o}^{i}-f_{b}^{i}N_{o}\right)}^{2}}{f_{b}^{i}N_{o}}$$ (1)

Where $N_{o}=\overset{K}{\underset{i=1}{\sum }}c_{o}^{i}$ is the total number of observations.

P-values are then computed using a $\chi^{2}$ distribution with *K*-1 degrees of freedom, where *K* is the number of events. False-discovery rate (FDR) correction is also applied to correct *p*-values for multiple testing. We computed statistical validation for classes with at least 20 observations.

### Computation of log-odds

Given a domain-specific (either Pfam or InterPro) observed frequencies $f_{o}$ (either the frequency of SRV types or Mondo categories) and a corresponding background distribution $f_{b}$ , we compute log-odd scores as follows:

$$LOGD=\log\frac{f_{o}}{f_{b}}$$ (2)

For avoiding numerical errors in the computation of the logarithm, we introduced pseudocounts when computing $f_{o}$ .

When appropriate, we report the median value of variations per protein, grouped according to the Pfam/InterPro domains, to highlight the central value of the distribution, independently of outliers.

In order to assess the range of variability of the computed values, we performed a bootstrap experiment by downsampling, with repetition, 80% of dataset 20 times and by computing the standard deviation of the resulting set of log-odds.

## Results

### The union data set

Our dataset is described in Table 1. When the union between Humsavar and ClinVar is considered (Union), it includes 75,145 variations (43,917 of which are pathogenic) in 3,605 genes. Pathogenic variations (LP/P) are linked to 5,223 diseases. Humsavar and ClinVar differently contribute to the Union data set; interestingly ClinVar contributes with a larger LB/B number of variations and a larger number of diseases to Union (Table 1, between brackets). When LP/P variations are annotated with OMIM or Mondo codes in both datasets, the overlap between the lists of associated diseases is 82.4%. Considering the 2,576 shared genes, the overlap of the associated diseases between ClinVar and Humsavar is 74.2% (Table 1).

**TABLE 1:** General description of the Union dataset.

	Humsavar	ClinVar	°Intersection	°Union
	#	#	#	#
Disease-associated genes	2,984 (408)*	3,197 (621)*	2,576	3,605
Variations in disease-associated genes	41,693 (25,035)*	50,110 (33,452)*	16,658	75,145
- Pathogenic	29,579 (17,371)*	26,546 (14,338)*	12,208	43,917
- Benign	12,114 (7,664)*	23,564 (19,114)*	4,450	31,228
Associated diseases^	3,898 (593)*	4,629 (1,324)*	3,305	5,223

°Intersection, °Union: Intersection and Union of Humsavar and ClinVar, respectively.

^Mondo IDs (5152) and OMIM (71).

*Between brackets: Exclusive items for each database, included in Union.

### Union genes and their disease association

The molecular function of the 3,605 genes in the Union dataset has been derived from the UniProt entries of their encoded proteins. We considered the annotation in terms of 30 high-level terms of the Molecular Function branch of the Gene Ontology^16^ (GO-MF) (Gene Ontology Consortium 2021) and of the Enzyme Commission numbers (EC) (Pundir et al., 2017). Some 38% of the dataset consist of enzymes: 1230 proteins are endowed with one or more EC number (Supplementary Table S1). Some 136 are annotated with a catalytic activity (GO:000382) and 15 are annotated as ATPases (GO:0016887) without EC number.

The other high-level GO-MF terms significantly over-represented in our dataset are GO:0140110 (transcription regulator activity, 277 genes), GO:0005198 (transporter activity, 239 genes), GO:0005198 (structural molecule activity, 159 genes), GO:0098772 (molecular function regulator activity, 135 genes), GO:0060089 (molecular transducer activity, 119 genes). GO:0005488 (binding) annotates 598 genes and the remaining high-level GO classes for MF account for a total of 76 proteins. Multiple high-level GO-MF terms are annotated for 308 genes and 313 genes lack GO-MF annotation.

**FIGURE 1:**
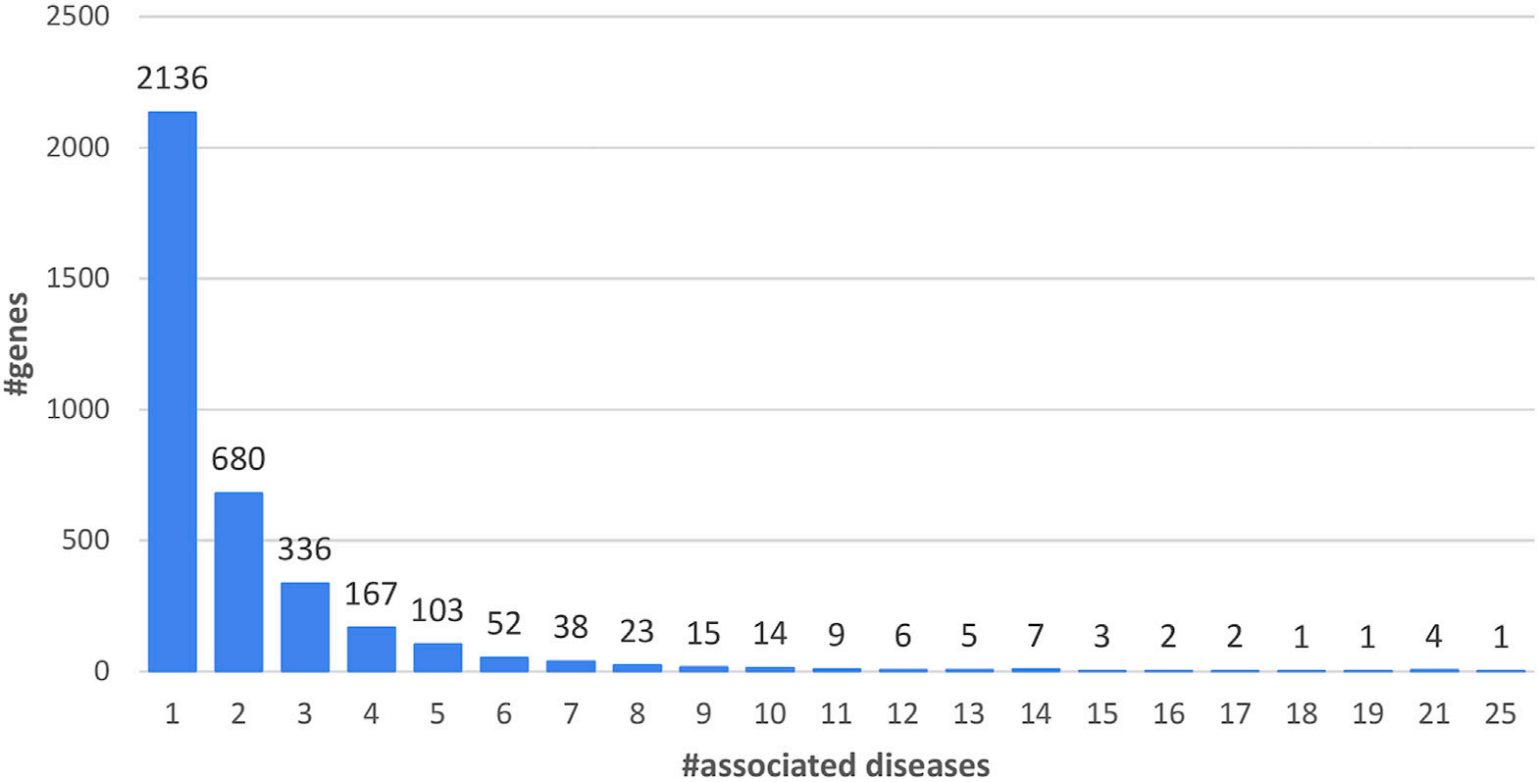
Distribution of Union genes as a function of the number of associated diseases (5,223 diseases) (Table 1).

Union genes are associated to diseases (Figure 1) and 59% of the genes are associated to one disease. 41% of the Union genes are associated to more than one disease. Genes associated with the highest numbers of diseases are Fibrillin, (FBN1, UniProt code: P35555), the GTPase KRas (KRAS, UniProt code: P01116), the Cellular tumor antigen p53 (TP53, UniProt code: P04637) and the Collagen alpha-1(II) chain (COL2A1, UniProt code: P02458), with 21 disease-associations. Prelamin-A/C (LMNA, UniProt code: P02545) is associated with 25 diseases.

**FIGURE 2:**
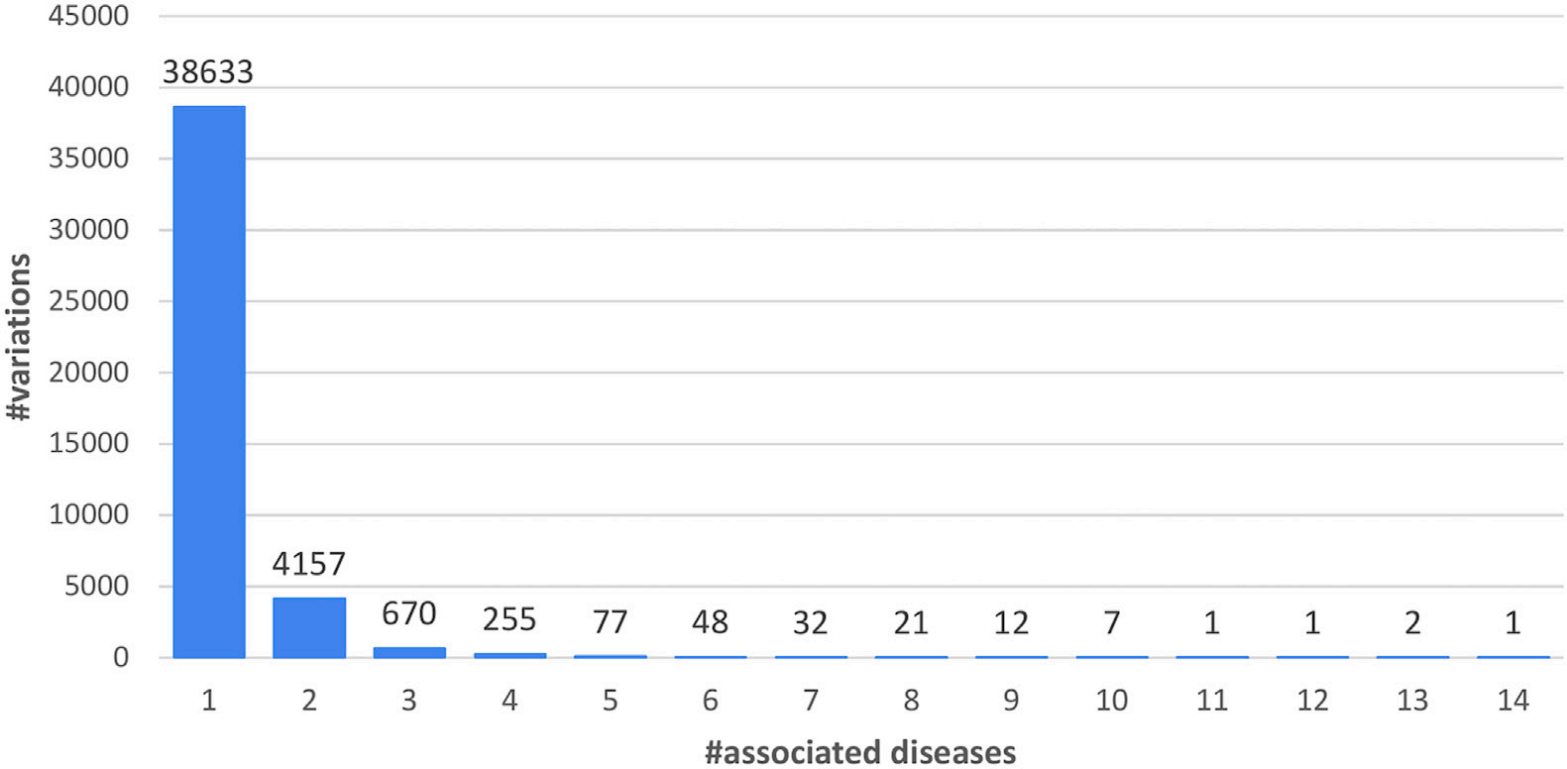
Distribution of the 43,917 LP/P variations in the Union data set as a function of the number of associated diseases (5,223).

Union variations are listed as a function of the number of associated diseases, as represented by Mondo IDs and 71 OMIM codes (Figure 2). 88% of the variations have only one disease-association. The variation associated with more diseases (14 in Figure 2) is P250R on FGFR3, the Fibroblast growth factor receptor 3 (UniProt code: P22607). Its variation is associated to the Muenke syndrome (MNKS), a condition characterized by coronal craniosynostosis, which affects the shape of the head and face, often with a decrease in the depth of the orbits and hypoplasia of the maxillae. Therefore, the variation, associated to 14 Mondo IDs, maps to 5 Mondo anatomical system categories (E, H, I, K, L; see Disease classification in Materials and Methods).

The distribution of diseases with respect to the number of genes and variations they are associated with are shown in Supplementary Figure S2, S3, respectively. They show that in our dataset most 4,595 out of 5,223 are monogenic and a large fraction (1,366) are associated with only one variation. In order to perform general and statistically significant analyses it is necessary to group genes, variations and diseases.

**FIGURE 3:**
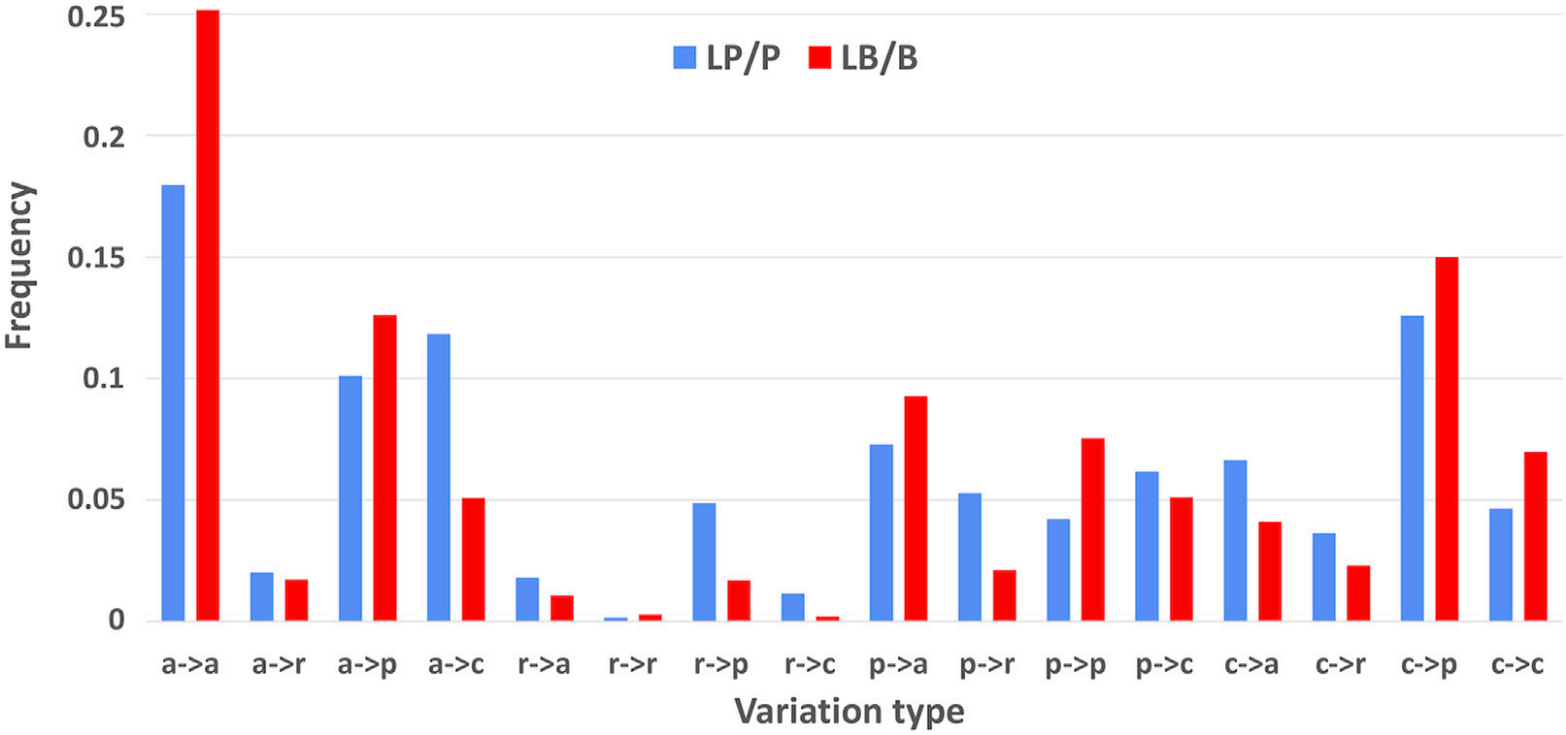
Frequency of variation types of the Union variations. Blue bars: LP/P variations; Red bars: LB/B variations. Labels are as follows: a, nonpolar; r, aromatic; p, polar; and c, charged.

For finding distinguished features among genes, variations, and diseases, we first grouped the disease related variations by variation types. To this aim, we firstly grouped residues according to their physicochemical properties, obtaining four major groups: nonpolar (GAVPLIM), aromatic (FWY), polar (STCNQH) and charged (DEKR) residues. We define a variation type in relation to the conservation or substitution of nonpolar (a), polar (p), aromatic (r) and charged (c) residues (Savojardo et al., 2019). Variations are then grouped into the 16 possible variation types, which allows to distinguish between residue substitutions which may affect protein stability and function based on the notion of being conservative or not, respectively. Results are in Figure 3 (and Supplementary Figure S1), which shows the different distribution of pathogenic versus benign variations in the different types. The variation types most frequently associated to diseases (LP/P) with respect to benign ones (LB/B), are a->c, c->a, p->r, r->p, c ->r, r ->c and r->a. Disease-related and benign variations have a different distribution and from now on we will focus on disease-related variations, being our goal to explore gene-disease association. The most abundant types of disease-related variations are nonpolar into nonpolar, polar, and charged, respectively, and charged into polar. These results agree with the more frequent variation types that we described as disease associated in a much smaller data set (Savojardo et al., 2019).

**FIGURE 4:**
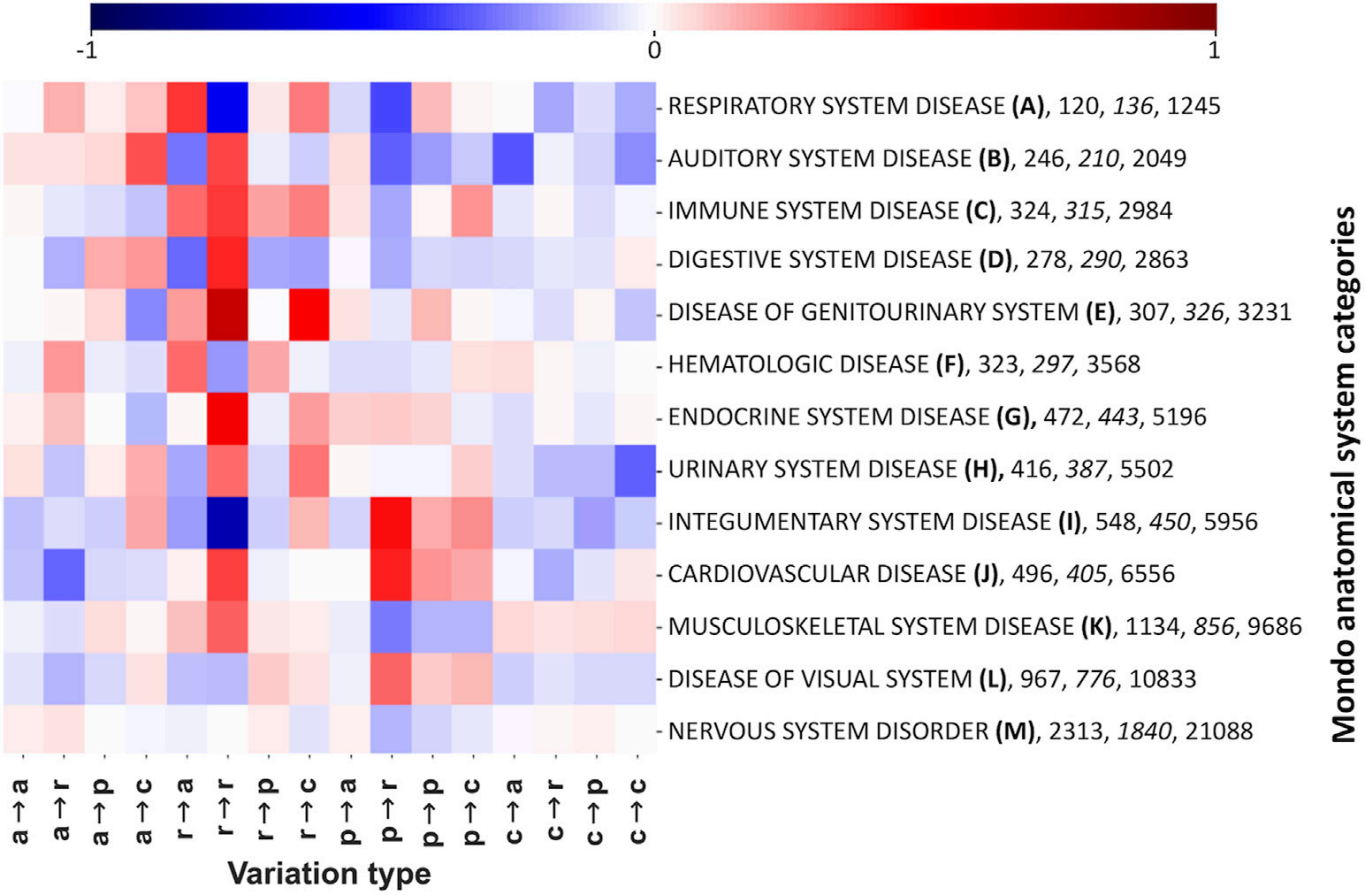
Log-odd scores of variation types associated to the different Mondo anatomical system categories. The heatmap shows the log-odd score of each variation type with respect to the corresponding LP/P background (shown in Supplementary Figure S1). For each Mondo category, we show the number of diseases, genes (italic) and disease related variations. In variation types, labels are as follows: a, nonpolar; r, aromatic; p, polar; and c, charged. The log-odd values are affected by a relative error lower than 5%, as estimated with a bootstrapping procedure. Statistical validation of the and resulting FDR-corrected *p*-values are reported in Supplementary Table S2.

The relationship among pathogenic variations associated to Mondo IDs and Mondo anatomical system categories is shown in the heatmap of Figure 4. Here we list as a function of the variation type, all the variations which are associated to the different Mondo anatomical system categories. For sake of clarity, we include the number of diseases in the set, the genes (italic) and the number of disease-related variations. The color-coded heat map indicates that for each category, the pattern of disease related variation types is different. A statistical validation of our findings is in Supplementary Table S2. In Figure 4, to better highlight over/under-representation, we show log-odds between each disease-type distribution and the background frequency of LP/P variations in the whole dataset.

### Pfam and InterPro coverage

In the following we take advantage of Pfam and InterPro coverage of each single gene to locate disease related variation types into structural and functional regions (Table 2). Pfam entries cover at least one pathogenic variant in 2,987 genes (83% of the 3,605 Union disease related genes, Table 1). Overall, 1,949 Pfam entries are identified in Union genes, including 32,575 pathogenic variations (74%). 1685 Pfams are endowed with an associated PDB structural domain. This analysis complements and confirms previous observation in a smaller data set (Savojardo et al., 2019; 2021b; 2021a).

**TABLE 2:** Pfam and InterPro coverage statistics.

	Pfam #	InterPro #
Union genes with at least one pathogenic variant in a Pfam and/or InterPro region	2,987 (83%)^a^	3,446 (96%)^a^
Domains covering pathogenic variants	1,949	5,357
Pathogenic variants in Pfam and/or InterPro regions	32,575 (74%)^b^	41,090 (94%)^b^
Benign variants in Pfam and/or InterPro regions	13,195 (42%)^c^	24,461 (78%)^c^

a Percentages computed with respect to the total number of diseases associated Union genes (3,605, Table 1).

b Percentages computed with respect to the total number of pathogenic variants (43,917, Table 1).

c Percentages computed with respect to the total number of benign variants (31,228, Table 1).

InterPro^17^, which integrates Pfam annotations with signatures taken from other member databases such as PROSITE, PRINTS and PANTHER, provides a larger number of functional regions. Indeed, with InterPro mapping we further enlarge the coverage at both gene and variation levels and can include some more 8,515 pathogenic variations in 459 genes (Table 2).

156 disease genes (4% of the total) do not have Pfam and/or InterPro domains including their pathogenic SRV positions. Finally, three SwissProt disease genes (Dentin sialophosphoprotein (UniProt: Q9NZW4), Uncharacterized protein FAM120AOS (UniProt: Q5T036) and Ribitol-5-phosphate xylosyltransferase 1 (UniProt: Q9Y2B1) do not have Pfam and/or InterPro signatures.

A complete list of the Pfam and InterPro regions, detailed for each gene, is reported in Supplementary Table S1. For each gene, we report the accession, the name, the functional annotation (EC, GO MF), the list of Pfam and InterPro domains, the number of pathogenic variations and associated diseases, the disease names and the associated Mondo disease anatomical system categories. Results highlight that the Pfam domain covering the highest number of disease related genes (62) is Pkinase (PF00069) while the domain mostly enriched in pathogenic variations (1,566) is Ion_trans (PF00520). Supplementary Table S1 lists also the results obtained with the InterPro coverage. Among the most abundant InterPro entries we found many conserved, binding, and active sites (as expected, being these important sites driving the gene/protein function). Some of them are within Pfam domains: e.g., the Homeobox_CS (IPR017970), included in the Homeodomain (PF00046) domain. This finding provides an additional specification of the most critical regions containing pathogenic variations.

### Distinctive patterns of pathogenic variation types within Pfam and InterPro regions

After structural and functional Pfam and IterPro gene mapping, we can analyze the relationship among variation types and diseases (grouped by Mondo anatomical system categories). With the concept of variation types (Figure 3), the 16 different SRV types can be associated to individual Pfam and InterPro (complete results are provided in Supplementary Table S3, which for Pfam and InterPro entry, include the number of genes, the number of LP/P variations, the frequencies of the variation type, the statistical validation and log-odds scores between domain-specific distributions and LP/P background frequency).

**FIGURE 5:**
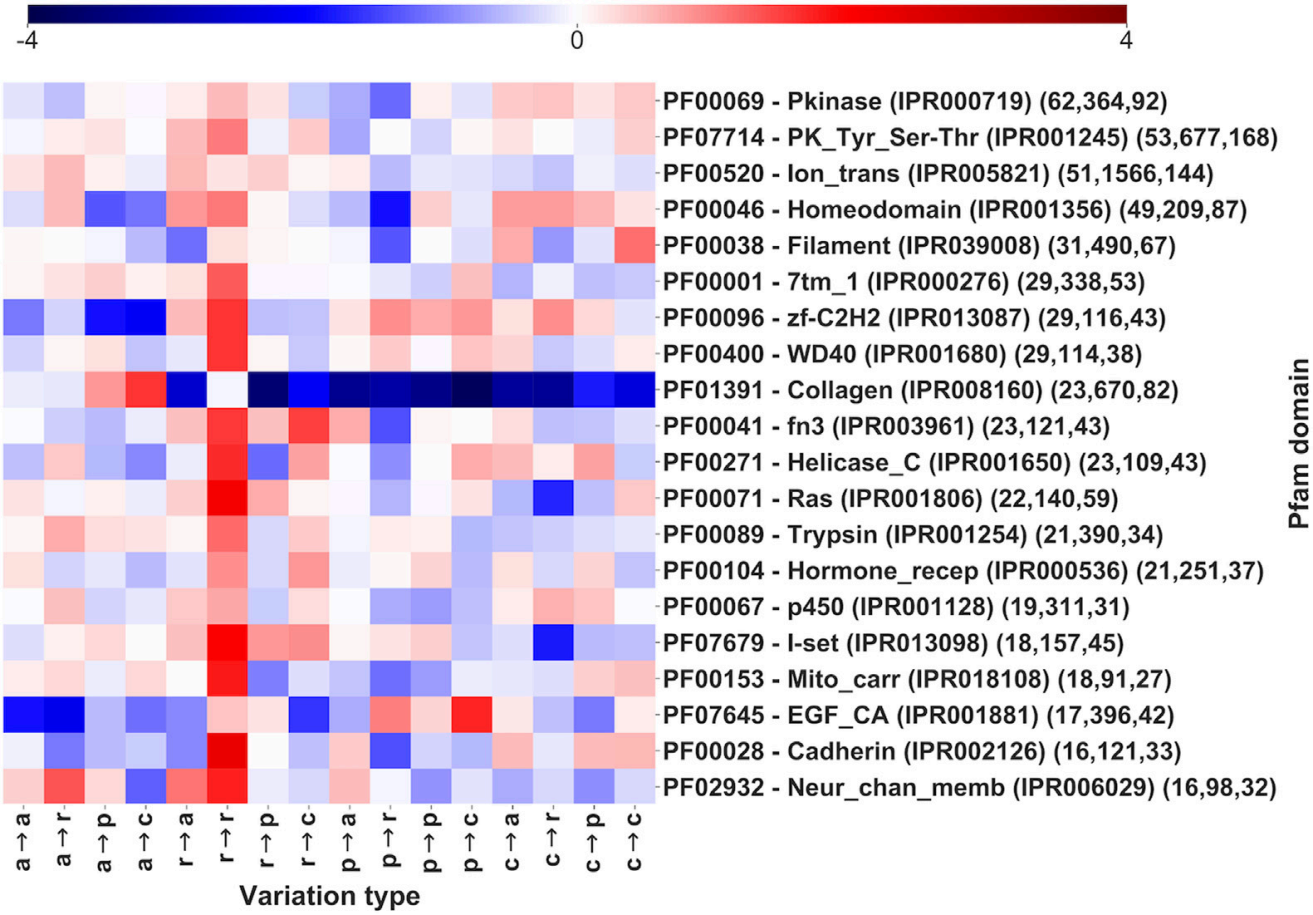
Log-odd scores of variation types in Pfam entries sorted by number of genes covered (the first 20, out of 1,940 Pfams, Supplementary Table S3). Log-odds are computed with respect to the whole dataset LP/P background (Figure 3). For each Pfam, the corresponding InterPro accession is also included. Numbers within parentheses report the number of genes, variations, and diseases, respectively. The log-odd values are affected by a relative error lower than 5%, as estimated with a bootstrapping procedure. Statistical validation and resulting FDR-corrected *p*-values for each Pfam entry are reported in Supplementary Table S3.

In Figure 5 we show the log-odd scores of pathogenic variation types for the 20 most populated Pfam domains (Figure 5). Pfams are sorted by the number of genes covered. For each domain, we report its Pfam accession and name with the number of genes and pathogenic SRVs covered, respectively (within parentheses). Overall, the 20 Pfams shown in Figure 5 cover 557 genes and 6,729 pathogenic SRVs, corresponding to 19 and 21% of the total number of Pfam-covered genes and SRVs, respectively (Table 2). In particular, genes covered by 6 out of 20 Pfams (p450, Pkinase, Ras, Trypsin, Helicase_C and PK_Tyr_Ser-Thr) are mainly associated with enzymatic activities, 2 (Homeodomain and zf-C2H2) occur in proteins performing transcription regulation activities (GO:0140110), 2 (Filament and Collagen) cover structural proteins (GO:0005488), 2 (Mito_carr and Ion_trans) are in transporters (GO:0005215), 1 (7tm_1) cover transducers (GO:0060089), 1 (Hormone_recep) is associated with proteins performing either transduction or transcription regulation activities, 1 (Neur_Chan_memb) is found in proteins associated to transport or transduction. The remaining 4 domains (fn3, EGF_CA, I-set, and Cadherin) have multiple associated functions and mainly act as mediators of interactions in proteins associated with a diverse range of functional activities.

Noticeably, the different Pfam domains show a distinctive variational pattern with significant deviations from the background distribution. Overall, our results confirm over a larger dataset, previous observations (Savojardo et al., 2021a). Statistical validation and resulting FDR-corrected *p*-values for each Pfam entry are also reported in Supplementary Table S3.

**FIGURE 6:**
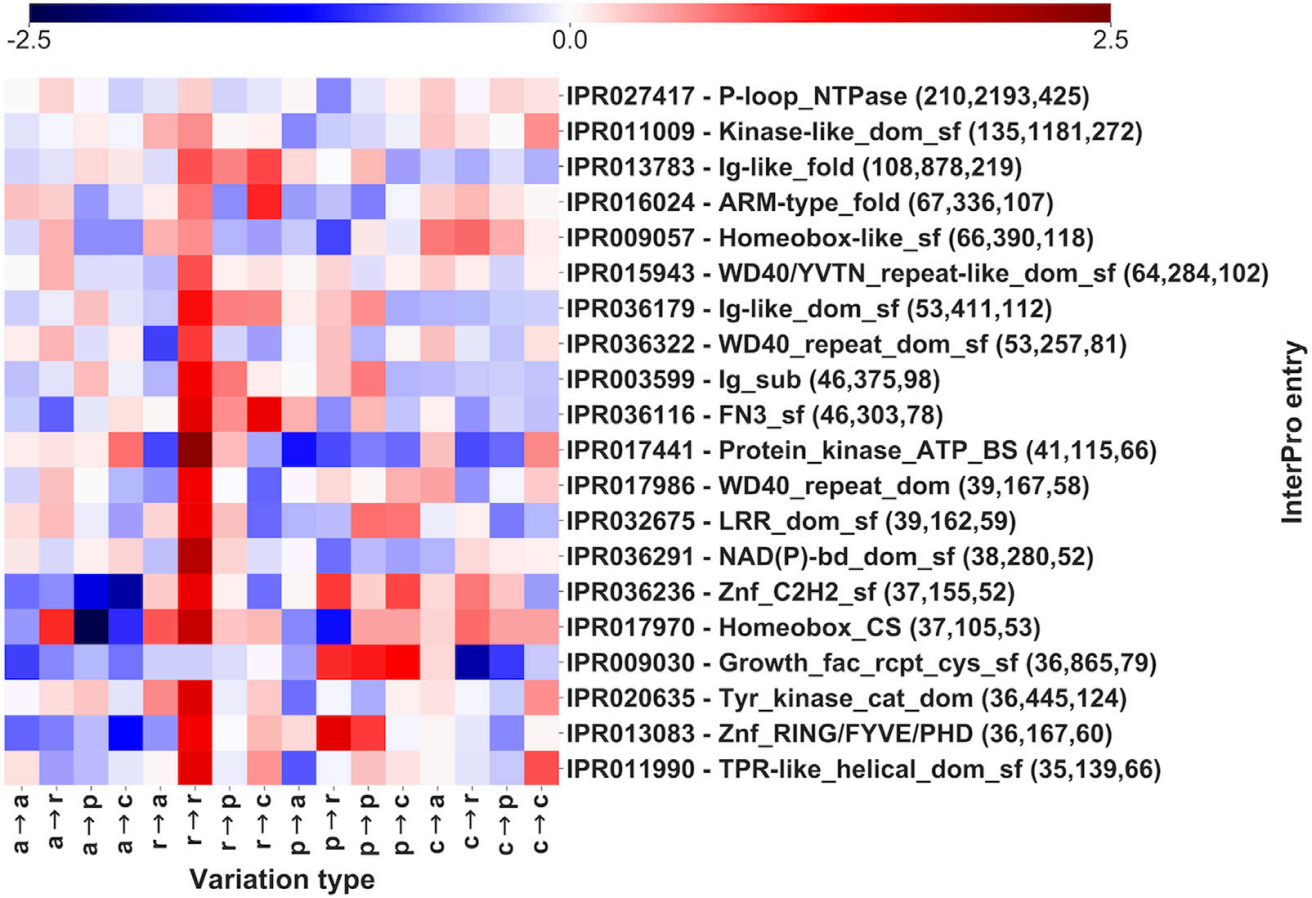
Log-odd scores of variation types for the first 20 InterPro entries (out of 5,357, Table 2), sorted by number of genes covered and not including Pfam signatures. Log-odds are computed with respect to the whole dataset LP/P background (Figure 3). Numbers in parentheses report, for each InterPro, the number of genes, of SRVs and of diseases, respectively. The log-odd values are affected by a relative error lower than 5%, as estimated with a bootstrapping procedure. Statistical validation and resulting FDR-corrected *p*-values for each InterPro entry are reported in Supplementary Table S3.

A similar analysis is performed for those InterPro regions that do not include Pfam domains (Figure 6 and Supplementary Table S3). The 20 InterPro entries in Figure 6 cover 836 genes and 9,208 pathogenic SRVs, corresponding to 24 and 22% of total number of InterPro-covered genes and SRVs, respectively (Table 2). Among the 20 InterPros, 9 cover proteins that are clearly associated to specific functions: 6 InterPros (Kinase-like_dom_sf, Znf_RING/FYVE/PHD, Protein_kinase_ATP_BS, Tyr_kinase_cat_dom, P-loop_NTPase and NAD(P)-bd_dom_sf) cover enzymes while 3 entries (Homeobox-like_sf, Homebox_CS and Znf_C2H2_sf) are associated to transcription factors. The other 11 InterPros are predominantly (not univocally) associated with proteins having different functions, including binding activities (Growth_fact_rcpt_cys_sf, WD40/YVTN_repeat-like_dom_sf, WD40_repeat_dom, LRR_dom_sf, Ig-like_dom_sf and WD40_repeat_dom_sf), molecular transducer activities (Ig-like_fold, FN3_sf) and 2 to enzymes (Ig_sub, TPR-like_helical_dom_sf).

Also in this case, different variational patterns can be observed for different InterPro entries.

### Associating Pfam/InterPro to Mondo anatomical system categories

In Figure 4, we established a relation between Mondo anatomical system categories and pathogenic variation types. In Figures 5, 6, we detailed the association among variation types and Pfam/InterPro regions in the different genes. For sake of generalization, an important question to answer is then to which extent Pfam and/or InterPro domains can be directly related to diseases grouped according to Mondo categories.

**FIGURE 7:**
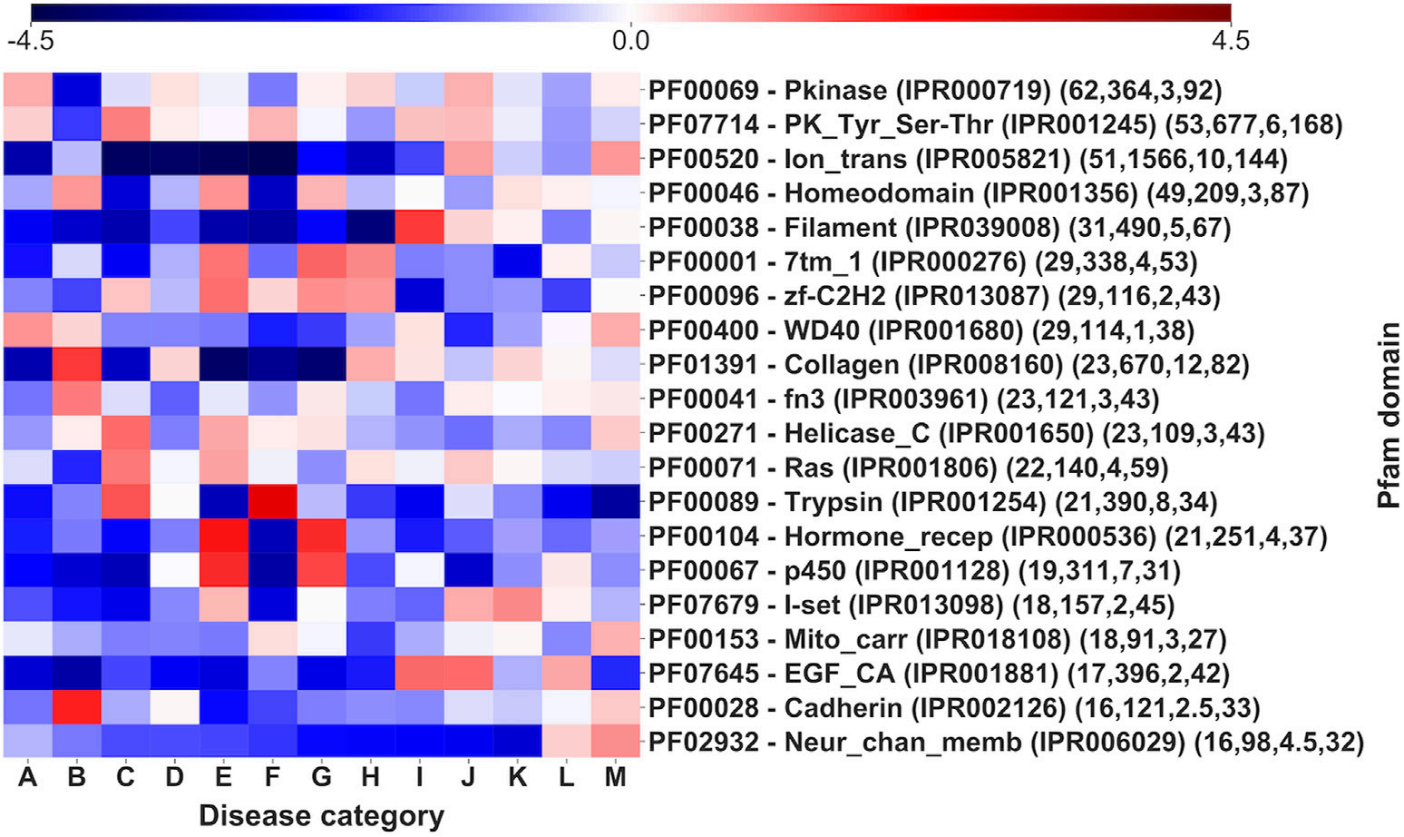
Log-odd scores for disease categories associated to different Pfam domains. Log-odds are calculated with respect to the whole-dataset background of disease categories (Supplementary Table S4). For each Pfam the corresponding InterPro accession is indicated. Numbers in parentheses report the number of genes, of SRVs, the median number of SRVs per gene and the number of diseases (for statistical validation see Supplementary Table S4).

Figure 7 shows log-odd scores for the disease Mondo categories associated to the 20 most populated Pfam domains (the full association with the 1,949 Pfam domains covering our Union set are listed in Supplementary Table S4, also including the background distribution frequency of disease categories in the entire set and the statistical validation results).

**FIGURE 8:**
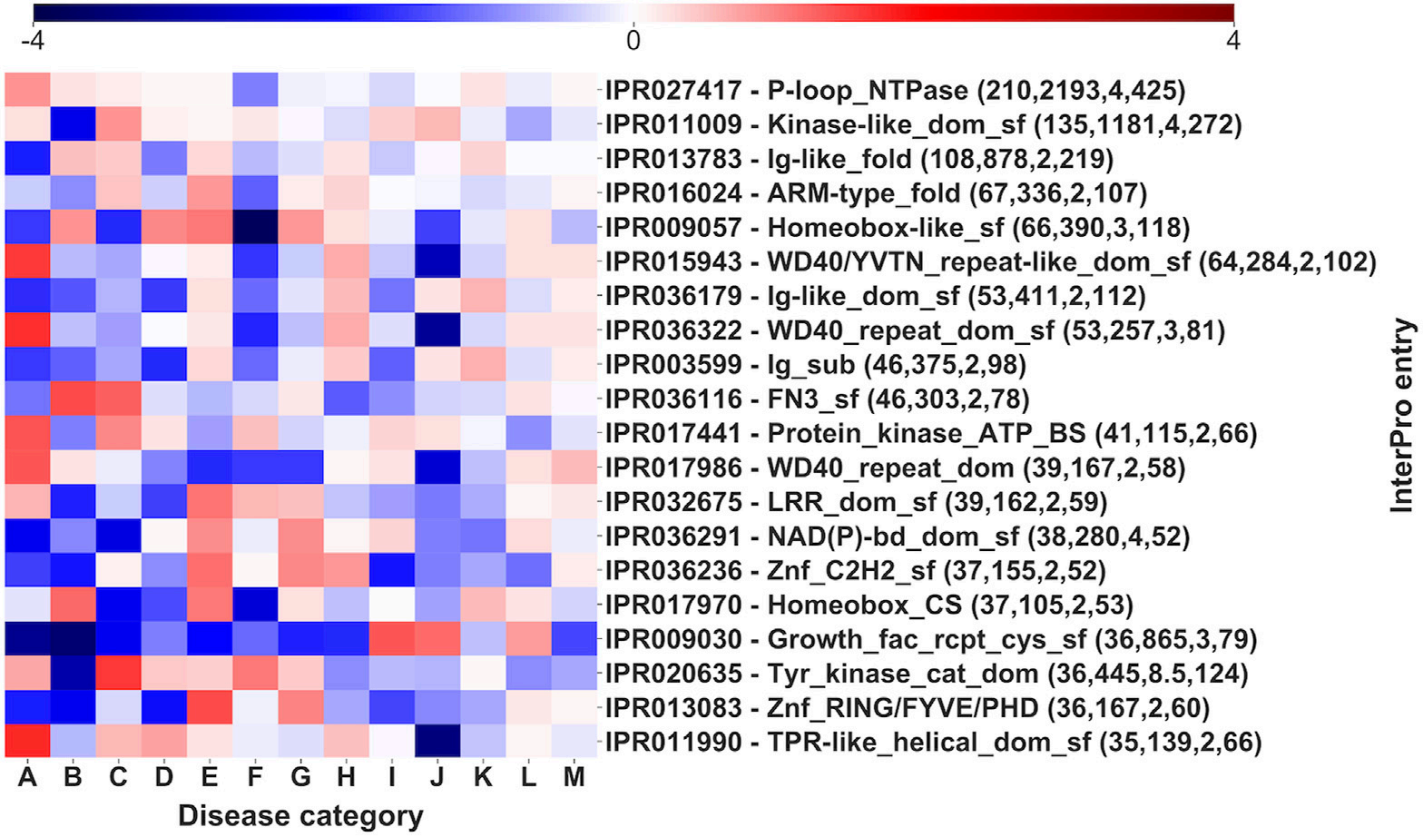
Log-odd scores for disease categories associated to different InterPro domains. Log-odds are calculated with respect to the whole-dataset background of disease categories (Supplementary Table S4). Numbers in parentheses report the number of genes, of SRVs, the median number of SRVs per gene and the number of diseases (for statistical validation see Supplementary Table S4).

Pfam domains are associated to multiple disease categories, as visible by comparing with the background signal. However, it is evident (Figure 7) that there is often one or more prevalent category/ies with an evident and significantly high log-odd score. For instance, in the case of Trypsin domain (PF00089), about 63% of the pathogenic variations associates to Hematologic diseases (F), a percentage significantly higher than the background frequency of this type of disease in the whole set (4%). Remarkably, these SRVs come from different genes (the median number of SRVs per gene for the Trypsin domain is 8). The same situation can be observed for other domains, like Ion_trans (PF00520), particularly enriched in neurological diseases (M). Finally, similar conclusions are obtained, when a similar heatmap is generated considering the relationship among Mondo anatomical system categories and InterPro regions that do not include Pfam signatures (Figure 8, reporting log-odd scores).

## Conclusions and perspectives

We investigate the relation between variants and diseases with the aim of finding possible descriptors for the association of genes carrying pathological variations and the corresponding diseases. To this aim we generated a dataset of variants with pathological and benign variations, union of the last releases of Humsavar and ClinVar (Table 1). Our focus are germline variations excluding somatic ones, whose associations to different types of cancers may require different ontologies.

We represent variations with variation types, which refer to their physicochemical properties. The distribution of disease-related and benign variation types of the union set is different (Figure 3). We therefore focused on the pathological variations, the carrying genes and the associated diseases, grouped into the corresponding Mondo anatomical system categories. We recognise that disease related variation types are specifically and significantly associated to different Mondo categories (Figure 4) and detailed the specificity by mapping variations into Pfam and InterPro regions. We find that these regions include most of the pathological variants (Table 2) and that the Pfam and InterPro mapping (Figures 7, 8) significantly associates to Mondo disease categories. A different confirmation on the stability of our results derives from the comparison with our previous results (Savojardo et al., 2021). The number of Pfams increases from 1,670 up to 1,949. When computing the Spearman’s correlation coefficient of the variation-type composition on the 247 Pfam domains that collect more than 20 variations in both samples, we obtain values ranging between 0.89 and 0.99. This indicates that the results obtained on the Pfam domains present in both analyses, are quite similar, despite the large difference in disease related variations (from 22,763 to 43,917) in the dataset size.

To our knowledge, the type of analysis that we propose is new and relies not only in associating domains to gene (Savojardo et al., 2021b), but also InterPro functional domains to them. Moreover, by showing that variation types show a statistically significant profile on specific domains, depending on the disease category, we indicate possible insights into the complex relationship among genes, variants, and associated diseases. Our final goal is to provide a mapping of the complex space relating variations, genes, and disease by means of gene structural and functional features. This can be useful for future algorithmic developments focusing on variant annotation. Possibly, new incoming data will be framed into our basic representation and will allow a better understanding of the mechanisms eliciting specific phenotypes linked to germline variations. However, before considering a prediction step, one major problem is at hand. Which is the real number of genes that are disease associated? We focused on germline variations for a very simple reason. The Monarch initiative and the Mondo ontology presently include the dataset we describe in this paper, namely 3,605 genes associated to 5,223 diseases. However, according to Pharos^18^, which includes DisGeNet^19^, the number of possible target genes is 20,412 and the number of associated diseases is currently 13,704, a large fraction of which is not characterized by reported variations in OMIM, Clinvar and Humsavar. Even worse, although Pharos includes Monarch, most of the common genes are associated also to different diseases. In this scenario, we believe that our findings, strengthened by this new analysis on a larger data set than before (Savojardo et al., 2021b), indicate a possible pattern of investigation.

## Data Availability

The original contributions presented in the study are included in the article/Supplementary Material, further inquiries can be directed to the corresponding authors.

## Footnotes

1
https://www.uniprot.org/docs/humsavar

2
https://www.ncbi.nlm.nih.gov/clinvar

3
https://mondo.monarchinitiative.org/

4
https://elixir-europe.org/

5
https://www.ebi.ac.uk/ols/ontologies/mondo

6
https://www.engreitzlab.org/resources/

7
https://pfam.xfam.org/

8
https://www.ebi.ac.uk/interpro/about/interpro

9
https://www.uniprot.org/docs/humsavar

10
https://www.ncbi.nlm.nih.gov/clinvar/

11
https://www.ebi.ac.uk/ols/ontologies/mondo

12
https://www.ebi.ac.uk/ols/index

13
http://obofoundry.org/ontology/mondo.html

14
ftp.ebi.ac.uk/pub/databases/Pfam/releases/Pfam33.1/proteomes/9606.tsv.gz

15
ftp.ebi.ac.uk/pub/databases/interpro/protein2ipr.dat.gz

16
http://geneontology.org/

17
https://www.ebi.ac.uk/interpro/

18
https://pharos.nih.gov/targets

19
https://www.disgenet.org
