# Supplementary figures and images for: Pathogenic variation types in human genes relate to diseases through Pfam and InterPro mapping
Source: Front Mol Biosci. 2022 Sep 16;9:966927. doi: 10.3389/fmolb.2022.966927 (PMC9523224; doi:10.3389/fmolb.2022.966927)

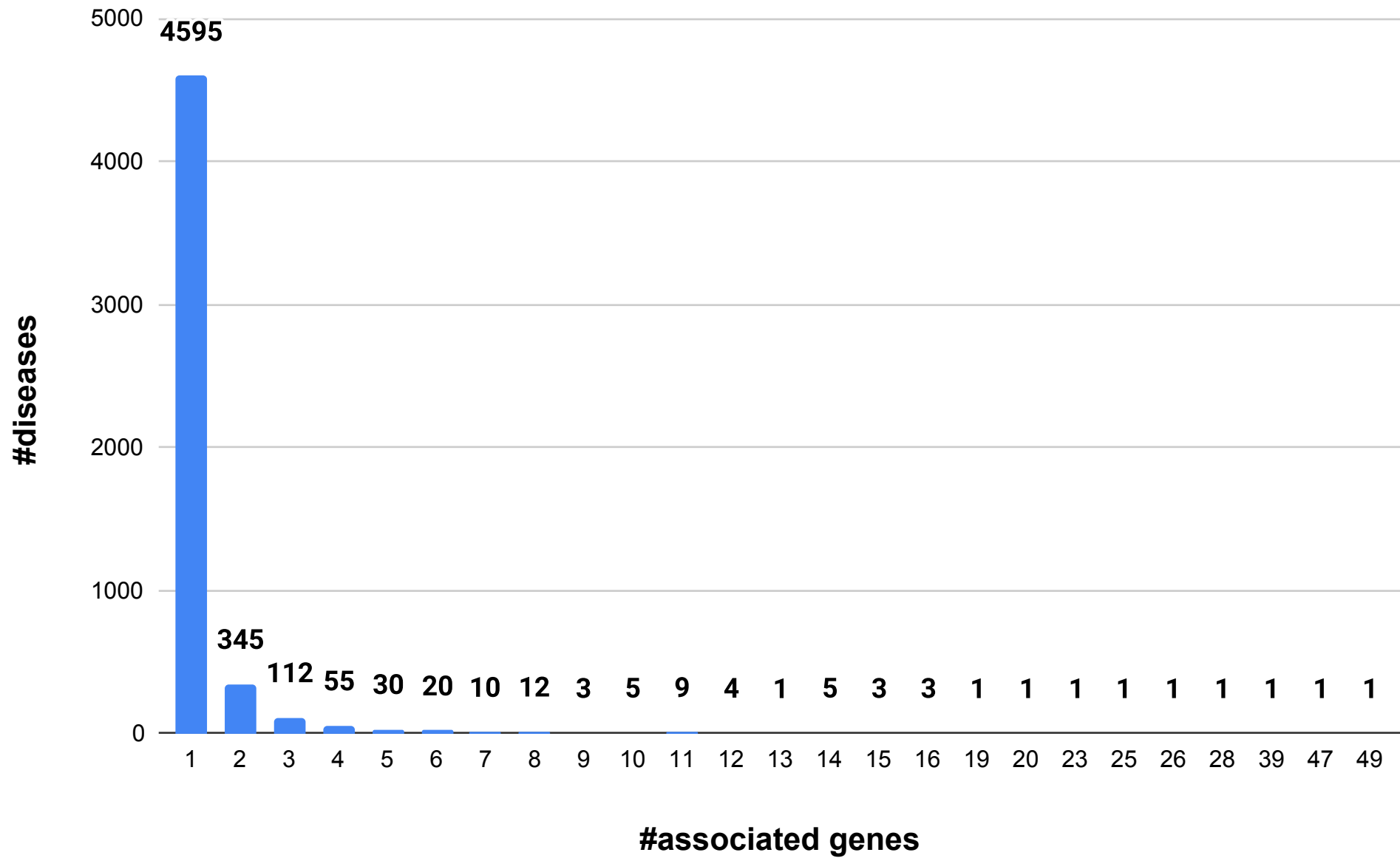

Supplement: Supplementary file 3 [file Image2.PDF]

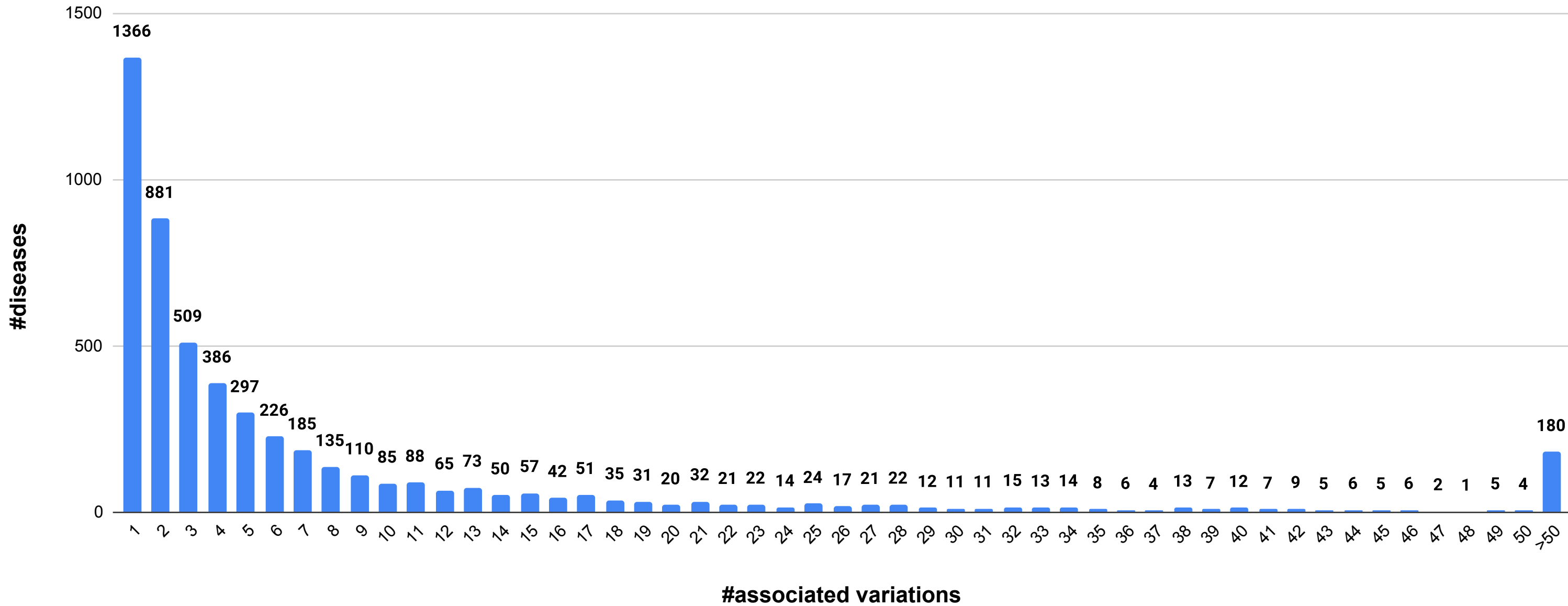

Supplement: Supplementary file 4 [file Image3.PDF]

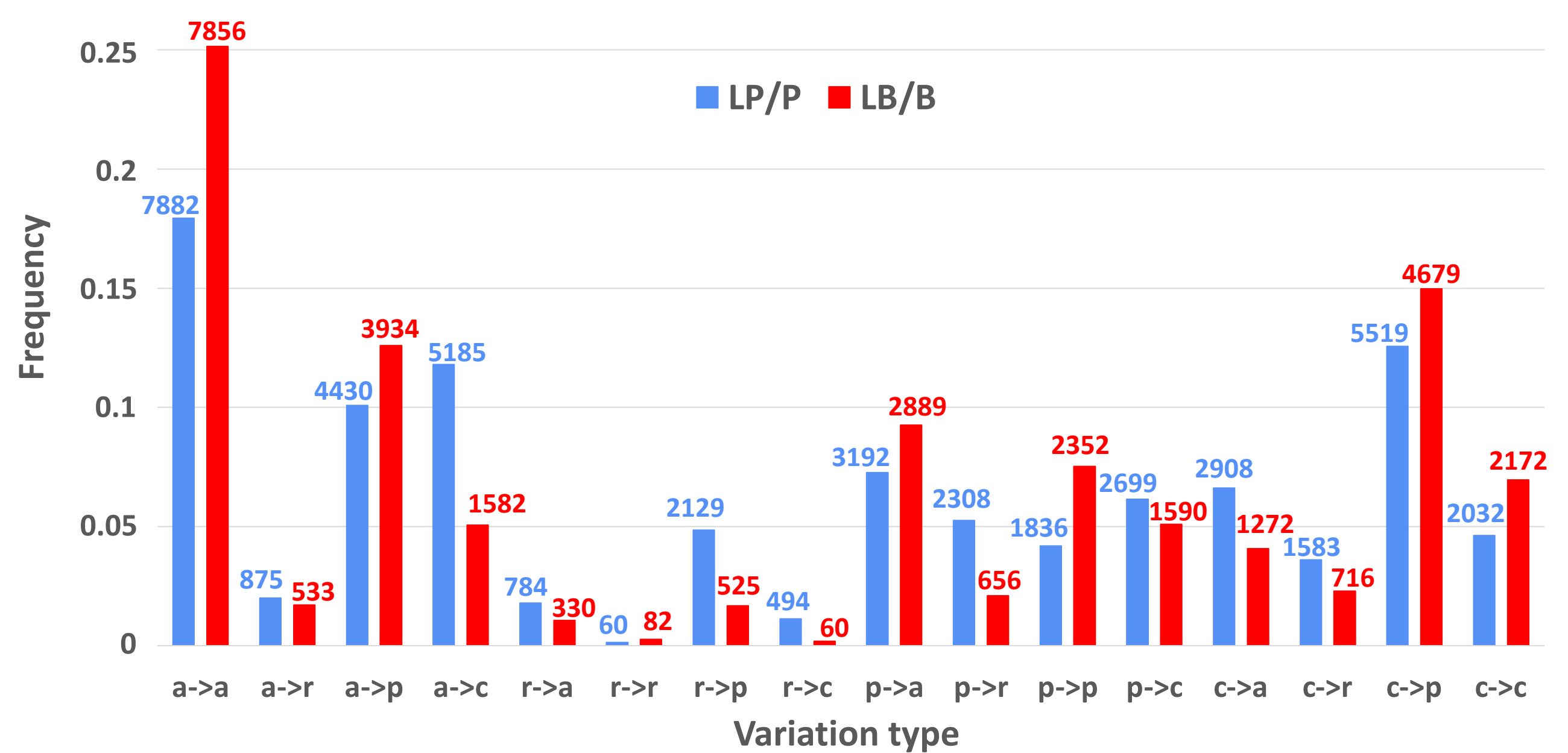

Supplement: Supplementary file 8 [file Image1.PDF]
