# Supplementary material for: Pathogenic variation types in human genes relate to diseases through Pfam and InterPro mapping
Source: Front Mol Biosci. 2022 Sep 16;9:966927. doi: 10.3389/fmolb.2022.966927 (PMC9523224; doi:10.3389/fmolb.2022.966927)
Supplement: Supplementary file 7 [file DataSheet1.DOCX]

**Supplementary Table 1.** The dataset of disease-related genes considered in this study. For each gene, we report: the gene symbol, the UniProt accession, the protein name, the functional annotation (EC, GO MF), the list of Pfam and InterPro domains, the numbers of pathogenic variations and associated diseases, the list of disease names and the associated Mondo disease anatomical system categories.

**Supplementary Table 2**. Distribution of diseases, genes and variations across the Mondo anatomical system categories. For each category we report the raw frequency of SRV types and corresponding log-odds computed with respect to the whole dataset background. * P-value assessing the statistical significance of the difference between the distribution of variation types for each Mondo anatomical system category with respect to the background distribution of pathogenic variations, as reported in Fig. 4 of the main text (compare each line corresponding to the anatomical system category with LP/P background). P-value is computed with the chi-squared test and corrected for multiple tests with the Bonferroni procedure. Anatomical categories with a corrected p-value lower that 0.01 are highlighted in bold.

**Supplementary Table 3**. Association between Pfam/InterPro and variation types. For each Pfam/InterPro we report the raw frequency of occurrence of each variation type, the log-odd score computed with respect to the whole dataset background (reported in the first row of the table) and p-values as obtained from statistical validation.

**Supplementary Table 4**. Association between Pfam/InterPro and Mondo anatomical system categories. For each Pfam/InterPro we report the raw frequency of occurrence of each disease category, the log-odd score computed with respect to the whole dataset background (reported in the first row of the table) and p-values as obtained from statistical validation.

**Supplementary Fig. 1.** Frequency of variation types of the Union variations. Blue bars: LP/P variations; Red bars: LB/B variations. Numbers on top of each bar report the absolute counts. Labels are as follows: a, nonpolar; r, aromatic; p, polar; and c, charged.

**Supplementary Fig. 2.** Distribution of Diseases as a function of the Union genes they are associated with

**Supplementary Fig. 3.** Distribution of Diseases as a function of the variations they are associated with
